# Use of Health and Well-Being Technology, Basic Psychological Needs, and the Mediating Role of Technological Identity in 6 European Countries: Prospective Longitudinal Survey Study

**DOI:** 10.2196/83054

**Published:** 2026-05-19

**Authors:** Moona Heiskari, Aki Koivula, Magdalena Celuch, Iina Savolainen, Teijo Osma, Atte Oksanen

**Affiliations:** 1 Faculty of Social Sciences Tampere University Tampere Finland; 2 Faculty of Social Sciences University of Turku Turku Finland

**Keywords:** digital health, health information technology, mobile apps, psychological needs, psychological theory, technological identity

## Abstract

**Background:**

Digital health technologies are increasingly used to monitor and improve personal health and well-being. Simultaneously, they can influence user behavior and self-understanding. As health technologies advance and are embedded in everyday life, understanding their broader psychological impacts, as well as the role of identity in shaping these outcomes, is crucial.

**Objective:**

This prospective longitudinal survey study examined how usage of health and well-being technologies predicts experiences of new technology-related basic psychological needs over time, reflecting the psychological outcomes of digital health technologies. Furthermore, we investigated whether technological social identity serves as a pathway through which health technology use is associated with basic needs.

**Methods:**

We used 3-wave survey data (2022-2024) collected from participants aged 18-75 years in Finland (n=1541), France (n=1561), Germany (n=1529), Ireland (n=1112), Italy (n=1530), and Poland (n=1533). Participants were recruited through a survey research panel, and follow-up data were collected from the same cohort. The sample was representative of the target populations by age (average age 46.79, SD 15.50 years) and gender (4304/8806, 48.88% male). The measure of digital health technology usage included smartphone health and well-being apps, well-being coaching apps, fitness trackers or watches, and smart rings. We applied a dynamic panel model within a structural equation modeling framework to examine both contemporaneous and lagged effects of technology use on the outcome variables—autonomy frustration, competence frustration, and relatedness satisfaction, measured with the Technology Effects on Need Satisfaction in Life scale. Mediation analysis was performed to assess whether social identification as a new technology user explained the relationship between technology use and need experiences.

**Results:**

Usage of health and well-being technologies was associated with higher technology-mediated relatedness (*β*=.14, 95% CI 0.11-0.18; *P*<.001). Autonomy frustration (*β*=.06, 95% CI 0.02-0.10; *P*=.003) and competence frustration (*β*=.06, 95% CI 0.02-0.10; *P*=.008) also demonstrated small but positive connections with technology use. We found no statistically significant differences across countries. Mediation analysis revealed that the relationships between technology use and psychological needs were largely explained by social identification as a technology user.

**Conclusions:**

Unlike most existing studies, this research focuses on the technology-related psychological effects of everyday health and well-being technologies and provides longitudinal and cross-national evidence of how they shape broader outcomes beyond health content and goals. The findings demonstrate that health technologies can both support users’ social needs and undermine their personal sense of agency. They also provide insights into the significance and dual-edged role of technology users’ social identity in transmitting these effects. The study highlights the central role of technology-related basic needs and identity processes in understanding the larger outcomes of rapidly advancing digital health technologies. It offers valuable insights for health technology designs and implementations that aim to enable need-supportive technology engagement.

## Introduction

### Overview

Personal health and wellness management has become increasingly digitized over the past 20 years [1-3], providing personalized health-related content, predictive analytics, and recommendations, which are increasingly artificial intelligence– and algorithm-based [4-7]. Understanding the psychological aspects of technology-mediated well-being management is critical in an era of individuals relying on advanced technologies in monitoring and improving their personal health, both in personal and clinical health care settings [8,9]. Particularly, there is limited knowledge about the psychological outcomes of human-technology interaction in the context of digital health technologies and the mechanisms underlying this relationship [2,10,11].

The aim of this study is to investigate how using health and well-being technologies affects the new technology-related basic psychological needs—autonomy frustration, competence frustration, and relatedness satisfaction—and whether in-group identification as a new technology user mediates these relationships. Technology-related autonomy reflects engaging with new technologies in ways that support one’s values and goals, whereas frustration arises when there is a sense of pressure to use technologies [12,13]. Technology-related competence refers to the extent to which new technologies help users feel capable and effective, whereas frustration reflects feelings of incapability and powerlessness [12,13]. Technology-enabled relatedness captures feeling socially connected to other people and communities through the use of new technologies [12]. Social identity processes are likely to shape these need-related experiences as technologies are increasingly embedded in users’ self-concepts and social group memberships. Building on this insight, we propose that in-group identification may help explain why health technology use supports or frustrates psychological needs.

### Well-Being Outcomes of Digital Health Technologies

Previous investigations on wearable and self-tracking technologies and mobile health apps have demonstrated positive associations between health technologies and well-being [14]. There is a significant amount of evidence that health application interventions are efficient in enhancing users’ quality of life and have positive effects in terms of reducing stress, anxiety, and depression [15-17]. Simultaneously, a growing body of research is studying the negative effects of self-tracking and their causes. For instance, self-quantification can decrease self-esteem, which may then reduce well-being [14]. Failing to achieve goals or engaging in social comparison when using health technology can also elicit negative emotions, such as guilt, anxiety, and stress [18]. The features of technologies, such as app notifications and human-like characteristics, might also cause stress, annoyance, and negative behavioral outcomes [19,20].

Health and well-being technology usage is not solely an individual practice; it also includes a clear social component in terms of offline and online interaction [21-23]. Social networking and sharing data with other users are part of health technologies, their tactics of behavior change, and how they motivate users [2,24,25]. Social networking can, however, yield varied outcomes. For example, reciprocal support in fitness apps may enhance autonomous engagement with health activities, while social recognition may foster compulsive behaviors [26].

### Basic Psychological Needs in the Use of Health Technologies

Self-determination theory (SDT) is a well-established theory that defines basic psychological needs of autonomy, competence, and relatedness as vital nutriments for well-being [27,28]. Extensive evidence has shown that the fulfillment of these needs is linked to well-being and happiness [29], while need thwarting predicts ill-being and negative outcomes [30]. Need satisfaction and need frustration are empirically distinct experiences, meaning that lack of need satisfaction does not necessarily equate to experiencing frustration and vice versa [13,27].

The motivation, engagement, and thriving in user experience model describes basic psychological needs as key mediators of the relationship between technology and well-being [12,31]. Existing research indicates that digital health technology can predominantly support users’ competence and autonomy needs, especially when it increases their knowledge, awareness, and control over healthy behaviors and lifestyles [21,32-34]. However, the available evidence suggests that the significance of feedback provided by wearable activity trackers diminishes over time, and that the initial feelings of inspiration may fade, leading to a lack of self-responsibility [21,34]. In terms of social connectedness, trackers can foster a sense of belonging and social support from family and friends who use the trackers together as well as from other users in online communities and platforms [21]. Prior research has shown that among users of online fitness communities, self-regulatory motivation—stemming from experiences of autonomy and competence—is particularly important for new users, whereas social motivation and enjoyment become more prominent for long-term users [35].

### Technological Social Identity as a Mediator of the Relationship Between Health Technology Use and Basic Needs

Personal IT identity has been shown to influence behavioral outcomes in technology use, and higher identification with technology has been found to predict broader and more explorative use [36,37]. Although the role of identity is increasingly recognized in research as human-technology interaction intensifies [38], evidence of the role of social identification in health technology use is lacking. The social identity approach emphasizes the human tendency to identify with social groups to which individuals perceive themselves as belonging, even when minimal cues of in- and out-group categorizations are present [39,40].

A large body of research evidence supports the fact that social identity predicts well-being outcomes, both positively and negatively, depending on the group’s qualities and status [41-43]. Studies on basic psychological needs have demonstrated similar associations [44,45]. One recent cross-national study demonstrated that in-group identification with technology users is systematically associated with basic psychological need outcomes, highlighting identification as a meaningful social context of technology use [46]. In terms of digital health technologies, early technology adopters can form a significant reference group with other early adopters and create a community—concrete or imaginary—among similar-minded people. When internalized as part of oneself, this group membership is prone to shape one’s interpretation and behavior when promoting uniformity in perception among group members, sharing values and norms, seeing oneself as similar to a group prototype, and comparing oneself to the group norm [47-49]. Thus, identifying as a technology user, which includes the norms and expectations one associates with the group, might influence the relationship and behaviors one has with health technology use, subsequently influencing the psychological outcomes.

As a mechanism linking health technology use and basic psychological needs, in-group identification as a technology user may support the user’s autonomy and mitigate their frustration as technology use is a part of one’s self-concept. Thus, aligning with group values and norms can promote internalized motivation to use technology [44]. In-group identification may enhance health technology users’ feelings of efficiency and mastery and decrease the competence frustration, as groups can help their members to feel more capable and personally in control [50]. Also, the group’s prototypical qualities can increase the individual’s sense of competence [49]. The relatedness of health technology users may be supported by identification with other technology users, particularly because it emphasizes belonging to a group of people with similar values, behaviors, and goals in terms of technology use [47,48]. As health technologies involve social components, identifying with other technology users can make an individual feel more connected and allow them to share experiences and obtain social validation from similar-minded individuals.

### This Study

The objective of this prospective longitudinal survey study was to investigate how health and well-being technology use is associated with technology-related basic psychological needs, and whether in-group identification as a technology user mediates this relationship. Our study design and hypotheses were preregistered in the Open Science Framework before the data analysis [51]. In the preregistration, we described SDT variables as need satisfaction. However, after revisiting the operationalization of our measures, we recognized that the autonomy and competence measures are more accurately conceptualized as need frustration. The hypotheses were therefore refined to reflect this operationalization. The fourth hypothesis was included after preregistration. The following hypotheses were proposed:

- H1: Health and well-being technology usage predicts lower new technology-related autonomy frustration.
- H2: Health and well-being technology usage predicts lower new technology-related competence frustration.
- H3: Health and well-being technology usage predicts higher new technology-related relatedness satisfaction.
- H4: The associations between the use of health and well-being technology and technology-related needs are mediated by in-group identification as a new technology user.

## Methods

### Participant Characteristics and Study Design

**Figure 1:**
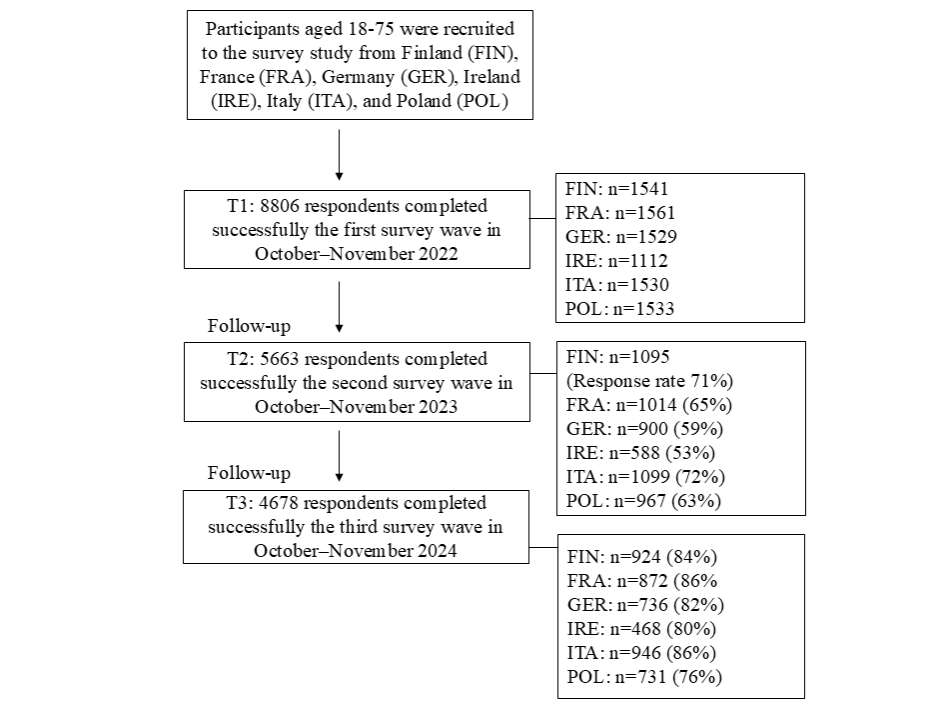
Study participant flowchart.

This study was reported following the American Psychological Association’s Journal Article Reporting Standards for Quantitative Research [52]. Our longitudinal survey data were collected as part of the Self & Technology research project, targeting participants aged 18-75 years from 6 European countries: Finland, France, Germany, Ireland, Italy, and Poland. The included European Union (EU) countries share major cultural and societal similarities but differ significantly when it comes to digitalization and the use of new technologies, making them suitable for investigating the role of health and well-being technologies in psychological functioning. The structured online survey was first conducted in October-November 2022, with a total sample size of 8806: Finland (n=1541), France (n=1561), Germany (n=1529), Ireland (n=1112), Italy (n=1530), and Poland (n=1533). Follow-up surveys were conducted in October-November 2023 and 2024 and targeted the same respondents who took part at time point 1. At T2, the response rate was 71% (1095/1541) in Finland, 65% (1014/1561) in France, 59% (900/1529) in Germany, 53% (588/1112) in Ireland, 72% (1099/1530) in Italy, and 63% (967/1533) in Poland. At T3, the response rate reached 84% (924/1095) in Finland, 86% (872/1014) in France, 82% (736/900) in Germany, 80% (468/588) in Ireland, 86% (946/1099) in Italy, and 76% (731/967) in Poland. The longitudinal sample flow, detailing participation and attrition across T1-T3 is presented in Figure 1.

The samples represent the age and gender demographics of the target populations in their respective countries [53]. Response rates were high at both follow-up times, 64% (5663/8806) of all the participants returned to the study at T2. In longitudinal studies, higher attrition typically occurs during the first follow-up, which was also visible in our data overall. At T3, a total of 53% (4678/8806) of T1 participants responded to the survey. Upon inspection, younger participants were more likely to drop out. This pattern of attrition is common in longitudinal studies and is often explained by changes in life circumstances over time. Missing data were examined with Little’s missing at completely random test and addressed in the analyses, as described in the Statistical Analyses section.

### Data Collection

Data collection was carried out by Norstat, a data solution provider. In the first study round, respondents were recruited from Norstat’s panel of voluntary research participants. Participants from the panel were invited via email to fill out the questionnaire about Europeans’ use of technology. Respondents to the first survey were reinvited via email to respond to the second and third study waves. The survey was originally designed in English and then expertly translated into the necessary languages by native speakers using the back-translation technique. Validated scales were used in each language when available. Data quality checks, such as patterned responses and attention checks, were conducted after each round of data collection to ensure data quality. Based on this, any questionable respondents were dropped from the final sample.

### Ethical Considerations

The study protocol was approved in 2022 by the Ethics Committee of the Tampere Region, which stated that the study did not involve ethical concerns (statement 115/2022). The survey respondents were provided with information about the study and the use of data for research purposes, highlighting that by completing the survey, respondents provided their informed consent to take part in the study. Respondents received compensation for their participation from Norstat. The company awards reward coins for each completed survey; the monetary value was approximately €2.60 (US $3.04) per survey in Finland, Poland, and Ireland, and €2.50 (US $2.93) in Germany, France, and Italy. These coins can be exchanged for gift cards, donated to charity, or used to enter prize draws. Participation in the study was voluntary, and the participants were informed about their right to withdraw their participation at any time without consequences. Participants were anonymous, and no information in the dataset allows for their identification. No individual participant can be identified from any part of the study materials. In addition, the study adheres to the EU’s General Data Protection Regulation.

### Measures and Covariates

Our measures of new technology-related autonomy, competence, and relatedness were adapted from the Technology Effects on Need Satisfaction in Life scale [12], which is based on the well-validated Basic Psychological Need Satisfaction and Frustration Scale [54]. We examined the degree to which respondents perceived new technologies to have influenced their sense of autonomy, competence, and relatedness in life overall. Ten statements were rated on a scale from 1 (does not describe me at all) to 7 (describes me completely). Four items measured sense of autonomy frustration (eg, “The new technologies intrude in my life”). One item was dropped to gain a better fit value in the confirmatory factor analysis conducted with structural equation modeling (SEM), resulting in a 3-item autonomy variable. Three items measured sense of competence frustration (eg, “Using the new technologies has made me feel insecure about my abilities”), and 3 items measured sense of relatedness satisfaction (eg, “Because of these new technologies, I feel closer to some others”). The full list of items is included in Multimedia Appendix 1. Higher scores for autonomy and competence express higher need frustration, while higher scores for relatedness indicate higher need satisfaction. The final scales ranged from 3 to 21. The reliability of the autonomy, competence, and relatedness measures varied from good to excellent at all 3 time points and across all 6 countries. See Multimedia Appendix 2 for the McDonald’s reliability figures of our measures.

As the independent variable, participants’ use of digital health and well-being technologies (wtech) was measured, including the use of smartphones and well-being apps, well-being coaching apps, fitness trackers and watches, and smart rings. The frequency of using these technologies was measured on a scale of 0-5 (0=do not use, 5=uses multiple times a day). We developed a sum variable to investigate the overall intensity of respondents’ usage of health and well-being technologies.

In-group identification with new technology users (IGI) was measured using a scale modified from the hierarchical model of in-group identification [55]. Out of the 5 components of in-group identification, we used the satisfaction dimension to measure the extent to which participants expressed positive feelings toward a group of new technology users and their membership in it. Positive feelings, conceptualized as satisfaction, are a direct and clear indicator of in-group identification that also indicate affective ties to the group and group-level self-investment [55,56]. Participants evaluated 4 statements (eg, “It is pleasant to be a user of new technologies”) on a scale of 1 (strongly disagree) to 7 (strongly agree). The reliability of the measure was excellent at all time points and across all countries.

For the control variables, we included the participants’ overall health status. We asked, “How would you evaluate your present health?” The participants were asked to provide an answer on a scale of 1 (very poor) to 7 (very good). As a straightforward and subjective assessment of one’s health, the self-rated health item effectively acts as a summary measure for different health dimensions, allowing respondents to select their own emphasis [57]. To assess self-esteem, we asked participants to rate the statement “I have high self-esteem” on a scale of 1 (not very true of me) to 7 (very true of me). The Single-Item Self-Esteem Scale used here has been found to be valid and has high convergent validity with longer global self-esteem scales in adult populations [58]. We also measured participants’ orientation for social comparison with a 6-item version of the Iowa-Netherlands Comparison Orientation Measure [59]. This scale is a shortened version of the 11-item version created by Gibbons and Buunk [60]. Finally, we asked study participants to evaluate the question “All things considered, how happy would you say you are?” on a scale of 1 (extremely unhappy) to 7 (extremely happy). Measuring the general sense of happiness with this single item has been found to be reliable and valid, as well as widely applicable, across different countries and cultures [61].

Sociodemographic variables included age, gender, and education. Age was measured in years. For gender, participants chose from the following options: “male,” “female,” or “other.” For the analysis, we created a dummy variable to reflect male vs other genders (0=not male gender, 1=male gender). For education level, participants were asked whether they had a university degree (at least a bachelor’s degree) and coded as 0 (no higher degree) and 1 (higher degree).

### Statistical Analyses

We performed dynamic panel models using the xtdpdml command in Stata (version 17; Stata Corp LLC) and conducted full information maximum likelihood estimation with standardized coefficients [62,63]. Within the SEM framework, this multilevel SEM method allowed us to study how the independent variable—use of health and well-being technology—influenced the dependent variables (ie, each technology-related need), while accounting for the previous level of need frustration or satisfaction. It further enabled us to estimate the lagged effects of health and well-being technology use. Simultaneously, with the within-level fixed effects, the dynamic panel model method allowed us to control for unobservable confounders [62,64]. We also included country-level dummy variables in our models to account for potential country differences. As the reference category, Finland was omitted, and the model included dummy variables for all other countries. The dummies were included as time-invariant covariates using the inv() command with xtdpdml. All models had a good fit based on traditional SEM thresholds (Multimedia Appendix 3), but these have limited relevance in dynamic panel models. For a more detailed analysis of country differences, we used random-effects within-between models to examine how within-person variation in the use of health technology is associated with basic needs across different countries.

Finally, we used a Monte Carlo simulation approach to examine whether IGI mediated the contemporaneous effect of health and well-being technology use (wtech) on the outcomes of technology-related autonomy, competence, and relatedness. For each outcome, we estimated the effect of wtech on IGI (path *a*) and the effect of IGI on the outcome variable (path *b*) using xtdpdml, which accounts for the lagged effects of the outcomes, individual-level fixed effects, and country differences. We then simulated 20,000 draws from normal distributions defined by the estimated coefficients and their SEs to generate a distribution of the indirect effect (*a* × *b*). From this distribution, we derived the mean indirect effect, 95% CIs, and 2-tailed *P* values.

Measurement invariance for the SDT and IGI factors was tested across countries using multigroup confirmatory factor analysis with maximum likelihood estimation using robust SEs in R software (version 4.5.2; R Foundation for Statistical Computing) with the *lavaan* package (0.6-20). We evaluated the overall fit of the initial model using widely applied fit indices and criteria, including the chi-square test of model fit (*χ*^2^), root-mean-square error of approximation (RMSEA)<.06, comparative fit index (CFI)<.95, and standardized root-mean-square residual (SRMR)<.08 [65]. Because the chi-square test is highly sensitive to large sample sizes and may inflate RMSEA, we relied on alternative fit indices and interpreted RMSEA with caution. When adding constraints and comparing nested models, we used the criteria of ΔCFI<0.01, ΔRMSEA<0.015, and ΔSRMR<0.03 to indicate support for each level of invariance [66].

### Missing Data

Missing data resulted from survey dropout across waves. We performed Little’s missing completely at random test on the continuous variables in the analytic models. The result indicated that the data were not missing completely at random (*χ*^2^_71_=879.42; *P*<.001). Therefore, we handled missing data with full information maximum likelihood, which uses all available data and provides valid estimates under the missing at random assumption. No auxiliary variables were included.

## Results

Table 1 presents the results of measurement invariance analyses. The initial configural model demonstrated good fit to the data (*χ*^2^_354_=2660.75; CFI=0.96; RMSEA 0.081, 90% CI 0.078-0.083; SRMR=0.062). RMSEA indicated acceptable fit given the large sample size, and the other fit indices suggested good fit, thus supporting further testing. Constraining the loadings across groups and comparing the fit indices supported metric invariance (ΔCFI=-0.001; ΔRMSEA=–0.004; ΔSRMR=0.001). Further constraining the intercepts (ΔCFI=–0.008; ΔRMSEA=0.004; and ΔSRMR=0.002) and residuals (ΔCFI=0.010; ΔRMSEA=0.003; and ΔSRMR=0.001) supported a strict level of invariance across countries.

**Table 1:** Measurement invariance results for basic psychological needs and in-group identification with new technology users across the countries.

	Chi-square (*df*)	Δ chi-square	*P* value (Δ chi-square)	CFI^a^	ΔCFI	RMSEA^b^ (90% CI)	ΔRMSEA	SRMR^c^	ΔSRMR
Configural	2660.75 (354)	N/A^d^	N/A	0.96	N/A	0.08 (0.08-0.08)	N/A	0.06	N/A
Metric	2831.07 (399)	117.85	<.001	0.96	–0.001	0.08 (0.07-0.08)	0.00	0.06	0.00
Scalar	3534.48 (444)	849.86	<.001	0.96	–0.008	0.08 (0.08-0.08)	0.00	0.07	0.00
Strict	3899.14 (509)	429.07	<.001	0.95	–0.010	0.08 (0.08-0.09)	0.00	0.07	0.00

^a^CFI: comparative fit index.

^b^RMSEA: root-mean-square error of approximation.

^c^SRMR: root-mean-square residual.

^d^N/A: not applicable.

Tables 2 and 3 present a descriptive summary of the variables included in this study, covering data from Finland, France, Germany, Ireland, Italy, and Poland. Across countries, the average score for technology-related autonomy frustration reached 8.50 at T1 and 7.61 at T3. A fixed-effects analysis showed a statistically significant decrease in within-person effects across all countries (*P*<.001). Competence frustration scores were 7.53 at T1 and 7.47 at T3, but did not show a statistically significant change. Relatedness satisfaction scored 9.45 at T1 and decreased to 8.11 at T3 (*P*<.001). The use of health and well-being technologies (wtech) showed a low level of 3.28 at T1 but moderately increased to 3.41 at T3 (*P*=.01). Finally, IGI scored, on average, 13.91 at T1 and decreased to 13.34 at T3 (*P*<.001). The fixed effects of the main variables are reported in detail in Multimedia Appendix 4.

**Table 2:** Descriptive statistics by country (Finland: n=1541; France: n=1561; and Germany: n=1529). Values for continuous variables are reported as mean, between-person SD (SDB), and within-person SD (SDW) across all time points. Age and categorical variables are reported from T1. Sample characteristics: Finland—male, 766 (47.71%); higher education, 413 (26.80%). France—male, 744 (47.66%); higher education, 572 (36.64%). Germany—male, 761 (49.77%); higher education, 450 (28.43%).

Variable		Finland				France				Germany		
	Range	Mean	SD_B_	SD_W_	Mean		SD_B_	SD_W_	Mean		SD_B_	SD_W_
Autonomy frustration	3-21	7.30	3.43	1.98	8.64		4.06	2.37	7.52		4.09	2.28
Competence frustration	3-21	6.62	3.59	2.02	8.35		4.20	2.55	6.87		4.07	2.25
Relatedness satisfaction	3-21	8.04	3.95	2.27	8.74		4.39	2.31	7.76		4.39	2.30
Wtech	0-16	3.21	3.10	1.23	2.27		3.00	1.22	2.48		3.11	1.17
IGI^a^	4-28	12.26	5.75	2.17	14.23		6.08	2.14	13.27		6.47	2.23
Self-rated health	1-7	4.68	1.24	0.56	4.80		1.29	0.53	4.57		1.41	0.56
Self-esteem	1-7	4.82	1.42	0.56	4.09		1.51	0.64	4.70		1.57	0.58
Social comparison	5-35	18.04	5.74	2.57	17.30		5.61	2.74	17.18		6.02	2.60
Happiness	1-7	4.89	1.11	0.52	4.67		1.18	0.57	4.66		1.27	0.71
Age (years)	18-75	46.35	16.34	N/A^b^	46.88		15.84	N/A	47.36		15.16	N/A

^a^IGI: in-group identification with new technology users.

^b^N/A: not applicable.

**Table 3:** Descriptive statistics by country (Ireland: n=1112; Italy: n=1530; and Poland: n=1533). Values for continuous variables are reported as mean, between-person SD (SDB), and within-person SD (SDW) across all time points. Age and categorical variables are reported from T1. Sample characteristics: Ireland—male, 540 (48.56%); higher education, 564 (50.72%). Italy—male, 749 (48.95%); higher education, 592 (38.69%). Poland—male, 744 (48.53%); higher education, 835 (54.47%).

Variable		Ireland				Italy				Poland		
	Range	Mean	SD_B_	SD_W_	Mean		SD_B_	SD_W_	Mean		SD_B_	SD_W_
Autonomy frustration	3-21	8.07	4.09	2.17	8.75		3.85	2.29	7.95		3.86	2.27
Competence frustration	3-21	6.85	3.99	2.26	8.25		4.19	2.54	7.77		4.17	2.38
Relatedness satisfaction	3-21	8.70	4.46	2.36	10.86		4.36	2.39	8.79		4.46	2.48
Wtech	0-16	3.74	3.34	1.23	3.39		3.48	1.56	3.64		3.78	1.63
IGI^a^	4-28	14.63	5.86	2.07	14.90		6.05	2.21	14.42		6.16	2.32
Self-rated health	1-7	4.91	1.31	0.53	4.97		1.21	0.55	4.58		1.25	0.55
Self-esteem	1-7	4.22	1.58	0.64	4.39		1.57	0.70	4.08		1.56	0.73
Social comparison	5-35	19.29	5.29	2.32	21.22		4.54	2.59	18.99		5.44	2.64
Happiness	1-7	4.87	1.25	0.69	4.65		1.23	0.59	4.74		1.25	0.64
Age (years)	18-75	46.77	14.50	N/A^b^	47.67		15.34	N/A	45.69		15.41	N/A

^a^IGI: in-group identification with new technology users.

^b^N/A: not applicable.

Table 4 reports the findings from Model 1, with separate analyses conducted for new technology-related autonomy frustration, competence frustration, and relatedness satisfaction. The wtech had a small but positive association with autonomy frustration (*β*=.06, 95% CI 0.02-0.10; *P*=.003), the CI not including zero, indicating a small but consistently positive population effect. Competence frustration similarly indicated a statistically significant positive effect (*β*=.06, 95% CI 0.02-0.10; *P*=.008). Conversely, wtech showed a positive association with relatedness satisfaction (*β*=.14, 95% CI 0.11-0.18; *P*<.001), the estimate remaining positive across the confidence bound. The effect of wtech on autonomy frustration, competence frustration, and relatedness satisfaction remained similar even when other factors were controlled (Model 4 in the Multimedia Appendix 5). With the lagged predictor, we found no evidence of a lagged effect of wtech use on autonomy, competence, or relatedness (Model 3 in the Multimedia Appendix 6).

**Table 4:** Dynamic panel models predicting the effect of health and well-being technology usage on technology-related basic psychological needs, presented without (Model 1) and with the mediator, in-group identification with new technology users (IGI; Model 2). Longitudinal time frame including T1-T3 (N=8806).

					Model 1									Model 2						
					*β* (SE)			*P* value			95% CI			*β* (SE)			*P* value			95% CI
**Autonomy frustration**																				
	Autonomy lag			0.06 (0.03)			.02			0.01 to 0.11			0.05 (0.02)			.03			0.01 to 0.10	
	Wtech			0.06 (0.02)			.003			0.02 to 0.10			0.01 (0.02)			.58			–0.03 to 0.05	
	IGI			N/A^a^			N/A			N/A			0.38 (0.02)			<.001			0.34 to 0.43	
	**Country (reference=Finland)**																			
		France	0.12 (0.01)			<.001			0.09 to 0.14			0.06 (0.01)			<.001			0.04 to 0.09		
		Germany	–0.01 (0.01)			.36			–0.04 to 0.01			–0.04 (0.01)			.005			–0.06 to –0.01		
		Ireland	0.04 (0.01)			.004			0.01 to 0.06			–0.01 (0.01)			.46			–0.04 to 0.02		
		Italy	0.12 (0.01)			<.001			0.10 to 0.15			0.06 (0.01)			<.001			0.03 to 0.08		
		Poland	0.04 (0.01)			.002			0.01 to 0.07			–0.01 (0.01)			.48			–0.04 to 0.02		
**Competence frustration**																				
	Competence lag			0.09 (0.03)			.003			0.03 to 0.15			0.09 (0.03)			.004			0.03 to 0.14	
	Wtech			0.06 (0.02)			.008			0.02 to 0.10			0.03 (0.02)			.16			–0.01 to 0.07	
	IGI			N/A			N/A			N/A			0.21 (0.03)			<.001			0.17 to 0.26	
	**Country (reference=Finland)**																			
		France	0.13 (0.01)			<.001			0.11 to 0.16			0.11 (0.01)			<.001			0.08 to 0.13		
		Germany	0.01 (0.01)			.40			–0.02 to 0.04			0.00 (0.01)			.86			–0.03 to 0.02		
		Ireland	0.01 (0.01)			.50			–0.02 to 0.03			–0.02 (0.01)			.22			–0.04 to 0.01		
		Italy	0.13 (0.01)			<.001			0.10 to 0.15			0.09 (0.01)			<.001			0.06 to 0.12		
		Poland	0.09 (0.01)			<.001			0.06 to 0.11			0.06 (0.01)			<.001			0.03 to 0.09		
**Relatedness satisfaction**																				
	Relatedness lag			0.04 (0.03)			.09			–0.01 to 0.09			0.00 (0.02)			.96			–0.04 to 0.03	
	Wtech			0.14 (0.02)			<.001			0.11 to 0.18			0.06 (0.02)			.001			0.02 to 0.09	
	IGI			N/A			N/A			N/A			0.65 (0.02)			<.001			0.61 to 0.69	
	**Country (reference=Finland)**																			
		France	0.09 (0.01)			<.001			0.06 to 0.11			0.00 (0.01)			.75			–0.02 to 0.02		
		Germany	–0.01 (0.01)			.63			–0.03 to 0.02			–0.05 (0.01)			<.001			–0.07 to –0.03		
		Ireland	0.04 (0.01)			.004			0.01 to 0.06			–0.04 (0.01)			<.001			–0.07 to –0.02		
		Italy	0.23 (0.01)			<.001			0.20 to 0.26			0.13 (0.01)			<.001			0.11 to 0.15		
		Poland	0.06 (0.01)			<.001			0.04 to 0.09			–0.02 (0.01)			.07			–0.04 to 0.00		

^a^N/A: not applicable.

**Figure 2:**
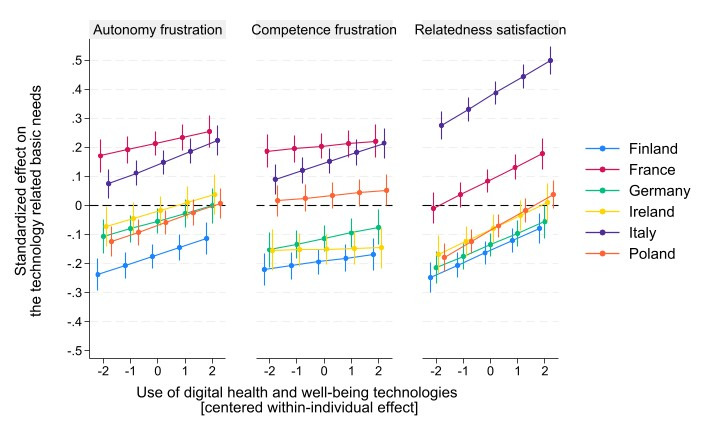
Within-person associations between health and well-being technology use and technology-related basic psychological needs across 6 countries (N=8806).

Model 1 illustrates the country differences of need frustration and satisfaction, with Finland acting as a reference point. Compared to Finland, autonomy was lower in all other countries (*P*<.01) except Germany. Competence was lower in France, Italy, and Poland (*P*<.001). The other countries, except Germany, showed higher relatedness than Finland (*P*<.01). Figure 2 depicts the country-level patterns in the within-individual (person-mean centered) association between the use of digital health and well-being technologies (wtech) and autonomy frustration (left panel), competence frustration (middle panel), and relatedness satisfaction (right panel). The lines represent predicted values derived from the random-effect within-between models, with other variables held constant. The x-axis reflects within-person deviations in technology use, and the y-axis shows standardized predicted levels of the respective need outcomes.

The results indicate that countries have broadly similar trends in their within-level associations between wtech and autonomy frustration and wtech and relatedness satisfaction. In most countries, predicted autonomy frustration increases as within-individual technology use increases. At the same time, predicted relatedness satisfaction increases consistently with higher levels of technology use across all 6 countries. In contrast, the pattern for competence frustration is more heterogeneous. A statistically significant positive association was observed in Italy (B=0.03, 95% CI 0.02-0.05; *P*<.001) and a marginally significant association in Germany (B=0.02, 95% CI 0.00-0.04; *P*=.07), whereas the association was negligible in the remaining countries.

Overall, in the random-effects within-between model (Multimedia Appendix 5), the main within-person effect of wtech on competence frustration became nonsignificant after including the country interaction terms. However, the reduction in the coefficient was approximately 15%, indicating that the association remained largely similar in magnitude and that cross-national variation in the within-person effect was modest. Moreover, formal tests of the cross-level interaction terms showed that none of the countries differed significantly from Finland in the strength of the within-individual association between technology use and autonomy frustration, competence frustration, or relatedness satisfaction (Multimedia Appendix 5). Thus, despite minor differences in predicted trends, the within-person associations did not significantly vary across countries relative to the reference category.

**Figure 3:**
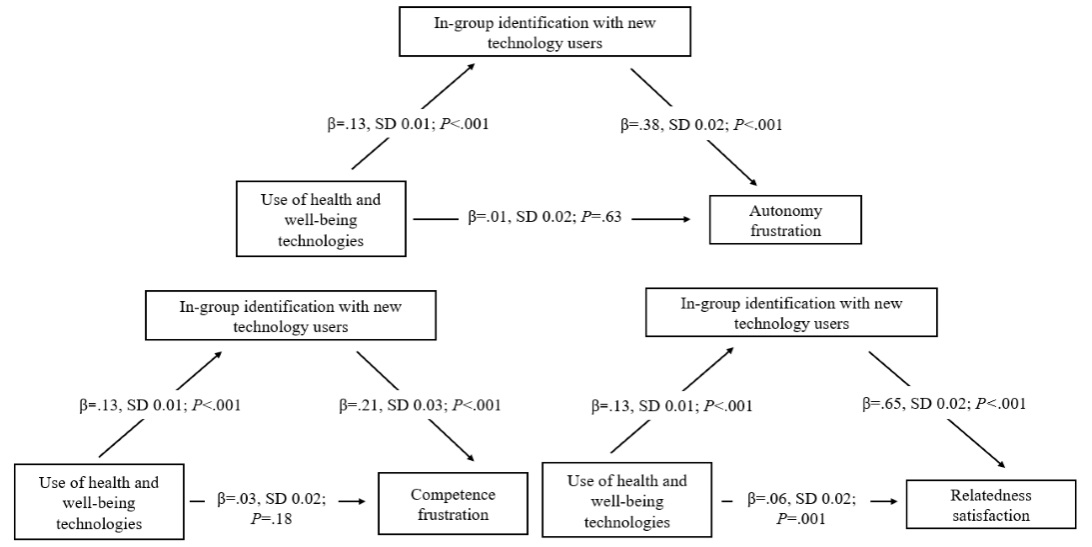
Mediation models showing the indirect association between health and well-being technology use and psychological needs through in-group identification with new technology users (N=8806).

Finally, Table 4 presents Model 2, which includes the IGI variable. Fully illustrated in Figure 3, mediation analyses showed that IGI is a significant factor explaining the association between wtech and technology-related need frustration for autonomy and competence and satisfaction for relatedness. Using a Monte Carlo simulation approach, we found that the indirect effect of IGI on autonomy frustration was positive, with the lower CI boundary above zero (*β*=.05, 95% CI 0.04-0.06; *P*<.001), accounting for 85.7% of the total effect. For competence frustration, the indirect effect was also positive, the CI excluding zero (*β*=.03, 95% CI 0.02-0.04; *P*<.001), accounting for 48.7% of the total effect. The indirect effect of IGI on relatedness was positive (*β*=.09, 95% CI 0.07-0.10; *P*<.001), accounting for 59.6% of the total effect.

## Discussion

### Main Findings

This prospective longitudinal survey study investigated the influence of health and well-being technology use, such as the use of smart rings, well-being apps, and fitness trackers, on basic psychological needs related to technology. Furthermore, it examined whether IGI mediated this association. Across 6 countries—Finland, France, Germany, Ireland, Italy, and Poland—we hypothesized that more intensive health and well-being technology usage would predict lower levels of new technology-related autonomy frustration (H1) and competence frustration (H2), and higher relatedness satisfaction (H3) over time. As we expected, higher health technology use was associated with higher relatedness. However, more intensive technology usage was associated with higher frustration with autonomy and competence. The mediation analysis revealed that IGI largely explained the outcomes of health and well-being technology use in terms of technological need frustration and satisfaction.

The 6 countries showed similar within-level associations between health technology use and autonomy and relatedness, with increased use generally linked to higher autonomy frustration and higher relatedness satisfaction. In terms of competence frustration, we found notable within-level associations only in Germany and Italy. However, with Finland as a reference country, no significant variation between countries was found in terms of the impact of health technology use on need frustration and satisfaction in general. Additionally, our investigation of the lagged effect of health technology use revealed that the contemporaneous effect of technology usage is more significant than the delayed impact on needs frustration and satisfaction.

### Theoretical and Practical Implications

Previous research has found positive impacts of health technologies on users’ health and well-being [14-16] but has also increasingly identified their adverse effects [18]. Our results indicate that health technologies’ overall effects on well-being are not solely determined by measurable outcomes. Rather, they are also closely tied to psychological processes that address individual needs. Based on SDT, our study offers a social psychological perspective on the role of health and well-being technology in responding to users’ basic psychological needs, showing that users may experience new technology-related autonomy and competence frustration despite simultaneously feeling that their relatedness needs are supported. Previous studies have demonstrated that digital health technologies can enhance users’ autonomy and competence when fostering a sense of control and providing information and resources [21,32-34]. Our study indicates that digital health and well-being technology users may experience their engagement with technologies as lacking self-endorsement and undermining their perceived sense of capability. This frustration of human agency is linked to the adverse effects associated with health technologies [18,31,67].

Focusing particularly on new technology-related needs, our study provides knowledge on technology’s role in psychological outcomes of health and well-being technologies that are increasingly sophisticated and incorporating artificial intelligence–driven features and advanced analytics [4-6]. This approach provides a novel understanding of technology-mediated processes of digital health management and their role on users’ basic psychological needs in life overall [12,31]. Understanding life-level impacts of new technologies is crucial, especially because health and well-being technologies are closely embedded into everyday routines, targeting users’ behavior and self-understanding. Overall, a more nuanced understanding of the psychological effects of human-technology interaction in digital health is needed, since most of the studies in the field have examined health technology acceptance and adoption [68].

Our study also investigated everyday health and well-being technologies more broadly, allowing us to identify general patterns, acknowledging that users may simultaneously use multiple health-related devices, applications, and platforms. This differs from the majority of health technology research that has examined the impacts related to tasks and behaviors with a focus on specific applications, interventions, and health conditions [14,69]. The longitudinal design also allowed for observing long-term trends, which is crucial in light of previous scholarship outlining the positive influence of digital health technologies may diminish over time [21,34].

This study also aimed to examine social identity–related processes underlying health technology use, and particularly how possessing a technology user’s identity contributes to well-being outcomes. IGI reflects a group-based identity that stems from the social categorizations and positioning of an individual among similarly minded technology users [39]. Our study tested whether this technological social identity mediates the association between well-being technology use and psychological needs. The social identity approach supports the notion that group membership affects the perception and behaviors of individuals [70]. In the context of technologies, norms and expectations of the technology users’ group are likely to shape users’ engagement with technologies, motivations, and future outcomes of health and well-being technology use. Our results indicated that the technology-enabled relatedness of health technology users is likely to be supported by IGI. Belonging to a group of people who share similar values, behaviors, and goals in terms of new technology seems to help satisfy relatedness in digital settings [43,44]. As health technologies involve social components, identifying with technology users might help an individual feel connected with other users and allow them to share experiences and receive social validation from like-minded individuals. This aligns with previous findings that users with a stronger personal IT identity seek more social engagement, as they are more willing to share their health information with others in health apps [71].

IGI did not support autonomy in health technology use; instead, it mediated higher autonomy frustration. Rather than promoting autonomous and self-directed technology use, technological social identity was linked to higher autonomy frustration, indicating more external pressure to use new technologies and a lack of personal control [12]. Similarly, technological identity mediated higher competence frustration, which suggests that instead of supporting mastery and efficiency in technology use, social technology user identity seemed to diminish technology-enabled competence feelings, connected to experiences of incapability and inadequacy [13]. Together, these results suggest that when technology is an integrated part of the social self, it can promote group norms and pressure an individual to use technology in a certain manner [37,42]. The results also suggest that when facing failure or negative comparisons to others—particularly prototypical or ideal technology users—satisfaction of autonomy and competence is diminished. This may be a potential downside for people with a stronger personal IT identity who use technology in a more intensive and explorative way [37,72]. IT identity has been similarly linked to embeddedness and enjoyment of technology use, but also to maladaptive emotions and problematic use [73]. Additionally, among new technology users, there may be more pressure to stay on track, master new technologies, and keep updated with the constant developments.

Our findings highlight the need to acknowledge the social identity perspective in health technology use, which typically involves social components and social gamification features [35,74,75]. Well-being supportive technology design should consider how to better enhance user agency in terms of competence and autonomy in technology use, but also take into account the group processes formed around technology use, including attitudes, norms, and expectations that shape how users experience and engage with technologies. Leveraging the social aspects of health and well-being technologies is a significant strength, as it can provide users with meaningful group memberships and a sense of belonging, thus supporting well-being beyond numerical tracking or goal achievement as well as long-term usage [22]. Future research should also investigate the factors shaping the overall psychological outcomes of health and well-being technology use in more detail.

### Strengths and Limitations

This study is based on longitudinal and cross-country evidence, both of which are needed in the field of digital health and well-being technology research. Our longitudinal and cross-national method enabled a nuanced investigation of the relationships between health and well-being technology use and the technology-related basic psychological needs, while also accounting for the role of IGI. However, this study is limited to self-reported data and to a European context; thus, any generalizations should be made with caution. Health and well-being technology use was operationalized through the use of smartphones, well-being apps, coaching apps, fitness trackers and watches, and smart rings. Hence, we did not inquire about the specific purposes for which these devices and apps were used. Future research should explore more detailed usage patterns and specific types of health technologies in relation to the basic psychological needs and technology user identity.

### Conclusion

This study offers novel insights into the psychological outcomes of everyday health and well-being technologies, focusing on new technology-related basic psychological needs. Our approach highlights that health technologies do not only passively transfer health content or collect data, but they also have a significant role in shaping how users experience the overall effects. The results showed that health technology use was associated with higher frustration of autonomy and competence, but also with higher satisfaction of relatedness. Our investigation also revealed that IGI largely transmitted these relationships. This finding advances the understanding of underlying factors that drive the psychological outcomes of health technology use and highlights the significance of technology users’ social identification. It particularly shows that this identification can support health technology users’ social needs but impair their sense of agency. The study provides important practical implications, highlighting how future health technology design needs to support users’ sense of personal control when adopting digital health technologies and fostering their sense of competence**.**

## Appendix

Multimedia Appendix 1 Measurement items.

Multimedia Appendix 2 Reliability figures, McDonald’s omega (ω).

Multimedia Appendix 3 Fit statistics for dynamic panel models.

Multimedia Appendix 4 Fixed effects showing temporal variation of the main variables between T1 and T3 (N=8806).

Multimedia Appendix 5 Country interaction effects between health technology use and autonomy and competence frustration, and relatedness satisfaction in the random-effects within–between model (N=8806).

Multimedia Appendix 6 Dynamic panel models predicting lagged effect of health and well-being technology usage on technology-related basic psychological needs, estimated with and without control variables (N=8806). Note: Models 1 and 2 are included in the main text.

## Abbreviations

CFI: comparative fit index
EU: European Union
IGI: in-group identification with new technology users
RMSEA: root-mean-square error of approximation
SDT: self-determination theory
SEM: structural equation modeling
SRMR: root-mean-square residual

## Data Availability

Data will be made available to the Finnish Social Science Data Archive after the end of the research projects. The data are available from the authors upon reasonable request.
