# Supplementary material for: Use of Health and Well-Being Technology, Basic Psychological Needs, and the Mediating Role of Technological Identity in 6 European Countries: Prospective Longitudinal Survey Study
Source: J Med Internet Res. 2026 May 19;28:e83054. doi: 10.2196/83054 (PMC13231114; doi:10.2196/83054)
Supplement: Multimedia Appendix 1 [file jmir_v28i1e83054_app1.docx]

| Effects of new technologies on the basic psychological needs in life based on the TENS-Life scale [12] |
| --- |
| *Autonomy frustration* |
| I spend more time on the new technologies than I feel I should. |
| The new technologies end up making me do things I don’t want to do. |
| The new technologies intrude in my life. |
| *Competence frustration* |
| Using the new technologies has made me feel insecure about my abilities. |
| Using the new technologies has made me feel less capable in my life. |
| Using the new technologies has lowered my confidence. |
| *Relatedness frustration* |
| Using the new technologies has helped me feel a greater sense of belonging to a larger community. |
| Using the new technologies has helped me feel close and connected with other people who are important to me. |
| Because of these new technologies, I feel closer to some others. |
| *In-group identification as a new technology user based on the hierarchical model of in-group identification [53]* |
| I am glad to be a user of new technologies |
| I think that users of new technologies have a lot to be proud of. |
| It is pleasant to be a user of new technologies. |
| Being a user of new technologies gives me a good feeling. |
