# Supplementary material for: Use of Health and Well-Being Technology, Basic Psychological Needs, and the Mediating Role of Technological Identity in 6 European Countries: Prospective Longitudinal Survey Study
Source: J Med Internet Res. 2026 May 19;28:e83054. doi: 10.2196/83054 (PMC13231114; doi:10.2196/83054)
Supplement: Multimedia Appendix 2 [file jmir_v28i1e83054_app2.pdf]

	T1	T2	T3	T1	T2	T3	T1	T2	T3
	Finland			France			Germany		
Autonomy frustration	0.82	0.83	0.81	0.84	0.85	0.84	0.85	0.85	0.85
Competence frustration	0.92	0.92	0.92	0.90	0.90	0.90	0.90	0.92	0.91
Relatedness satisfaction	0.89	0.89	0.91	0.89	0.92	0.92	0.89	0.91	0.92
IGI	0.94	0.95	0.94	0.93	0.94	0.95	0.94	0.94	0.94
SCO	0.85	0.85	0.85	0.83	0.84	0.85	0.83	0.83	0.85
	Ireland			Italy			Poland		
Autonomy frustration	0.84	0.81	0.81	0.81	0.85	0.85	0.81	0.83	0.83
Competence frustration	0.92	0.94	0.94	0.93	0.92	0.92	0.93	0.94	0.94
Relatedness satisfaction	0.90	0.92	0.93	0.90	0.92	0.92	0.92	0.94	0.95
IGI	0.94	0.93	0.94	0.95	0.96	0.96	0.93	0.94	0.94
SCO	0.80	0.81	0.81	0.80	0.80	0.78	0.82	0.84	0.83
	Total								
Autonomy frustration	0.83	0.84	0.83						
Competence frustration	0.92	0.92	0.92						
Relatedness satisfaction	0.90	0.92	0.93						
IGI	0.94	0.95	0.95						
SCO	0.82	0.83	0.84						
