# Supplementary material for: Use of Health and Well-Being Technology, Basic Psychological Needs, and the Mediating Role of Technological Identity in 6 European Countries: Prospective Longitudinal Survey Study
Source: J Med Internet Res. 2026 May 19;28:e83054. doi: 10.2196/83054 (PMC13231114; doi:10.2196/83054)
Supplement: Multimedia Appendix 3 [file jmir_v28i1e83054_app3.docx]

| Autonomy frustration models |  | Model 1 | Model 2 | Model 3 | Model 4 | Model 5 |
| --- | --- | --- | --- | --- | --- | --- |
|  | RMSEA | 0.000 | 0.000 | 0.000 | 0.000 | 0.000 |
|  | BIC | 193225.13 | 215448.09 | 213678.66 | 367628.23 | 387918.38 |
|  | AIC | 192807.23 | 214860.19 | 213175.75 | 365935.35 | 386062.59 |
|  | CFI | 1.000 | 1.000 | 1.000 | 1.000 | 1.000 |
|  | TLI | 1.00 | 1.002 | 1.002 | 1.002 | 1.002 |
|  | CD | 0.670 | 0.712 | 0.670 | 0.668 | 0.668 |
| Competence frustration models |  | Model 1 | Model 2 | Model 3 | Model 4 | Model 5 |
|  | RMSEA | 0.000 | 0.005 | 0.000 | 0.006 | 0.006 |
|  | BIC | 193690.31 | 216448.276 | 214400.98 | 368375.92 | 388858.18 |
|  | AIC | 193272.41 | 215860.371 | 213898.08 | 366683.04 | 387002.39 |
|  | CFI | 1.000 | 1.000 | 1.000 | 0.999 | 0.999 |
|  | TLI | 1.002 | 0.999 | 1.002 | 0.997 | 0.996 |
|  | CD | 0.639 | 0.653 | 0.641 | 0.643 | 0.644 |
| Relatedness satisfaction models |  | Model 1 | Model 2 | Model 3 | Model 4 | Model 5 |
|  | RMSEA | 0.000 | 0.000 | 0.000 | 0.000 | 0.000 |
|  | BIC | 190974.00 | 208896.34 | 210972.78 | 365157.30 | 385024.38 |
|  | AIC | 190556.09 | 208308.44 | 210469.88 | 363464.42 | 383168.58 |
|  | CFI | 1.000 | 1.000 | 1.000 | 1.000 | 1.000 |
|  | TLI | 1.000 | 1.001 | 1.000 | 1.003 | 1.003 |
|  | CD | 0.746 | 0.857 | 0.744 | 0.750 | 0.748 |
