# Supplementary material for: Use of Health and Well-Being Technology, Basic Psychological Needs, and the Mediating Role of Technological Identity in 6 European Countries: Prospective Longitudinal Survey Study
Source: J Med Internet Res. 2026 May 19;28:e83054. doi: 10.2196/83054 (PMC13231114; doi:10.2196/83054)
Supplement: Multimedia Appendix 4 [file jmir_v28i1e83054_app4.pdf]

	Finland			France			Germany		
	B (SE)	*P*	95% CI	B (SE)	*P*	95% CI	B (SE)	*P*	95% CI
Autonomia frustration	-0.47 (0.12)	<. 001	-0.70 to -0.23	-0.59 (0.15)	<. 001	-0.89 to -0.30	-1.36 (0.16)	<. 001	-1.66 to -1.05
Competence frustration	0.06 (0.12)	0.649	-0.18 to 0.29	-0.25 (0.16)	0.126	-0.56 to 0.07	-0.29 (0.16)	0.067	-0.60 to 0.02
Relatedness satisfaction	-1.54 (0.13)	<. 001	-1.80 to -1.28	-0.82 (0.14)	<. 001	-1.10 to -0.53	-1.30 (0.16)	<. 001	-1.61 to -0.99
Wtech	0.17 (0.07)	0.021	0.03 to 0.32	0.17 (0.08)	0.024	0.02 to 0.33	0.09 (0.08)	0.290	-0.07 to 0.25
IGI	-1.00 (0.13)	<. 001	-1.26 to -0.75	-0.45 (0.13)	0.001	-0.71 to -0.18	-0.84 (0.16)	<. 001	-1.14 to -0.53

	Ireland			Italy			Poland		
	B (SE)	*P*	95% CI	B (SE)	*P*	95% CI	B (SE)	*P*	95% CI
Autonomia frustration	-0.78 (0.19)	<. 001	-1.16 to -0.40	-0.49 (0.14)	<. 001	-0.75 to -0.22	-0.79 (0.16)	<. 001	-1.10 to -0.49
Competence frustration	-0.03 (0.20)	0.890	-0.43 to 0.37	0.16 (0.15)	0.288	-0.14 to 0.46	0.02 (0.17)	0.899	-0.30 to 0.35
Relatedness satisfaction	-1.20 (0.21)	<. 001	-1.62 to -0.79	-0.83 (0.14)	<. 001	-1.10 to -0.55	-1.17 (0.17)	<. 001	-1.50 to -0.83
Wtech	-0.01 (0.11)	0.937	-0.23 to 0.21	0.09 (0.09)	0.346	-0.09 to 0.27	0.00 (0.11)	0.970	-0.22 to 0.23
IGI	-0.71 (0.19)	<. 001	-1.07 to -0.34	-0.54 (0.13)	<. 001	-0.80 to -0.29	-0.87 (0.16)	<. 001	-1.19 to -0.56
