# Supplementary material for: Use of Health and Well-Being Technology, Basic Psychological Needs, and the Mediating Role of Technological Identity in 6 European Countries: Prospective Longitudinal Survey Study
Source: J Med Internet Res. 2026 May 19;28:e83054. doi: 10.2196/83054 (PMC13231114; doi:10.2196/83054)
Supplement: Multimedia Appendix 5 [file jmir_v28i1e83054_app5.docx]

| Autonomy frustration | M1 | | | | | M2 | | | | |
| --- | --- | --- | --- | --- | --- | --- | --- | --- | --- | --- |
| variables | B | SE | *P* | 95 % CI | | B | SE | *P* | 95% CI | |
| w_wtech | 0.13 | 0.02 | <.001 | 0.10 to 0.17 | | 0.14 | 0.04 | 0.001 | 0.05 to 0.22 | |
| b_wtech | 0.34 | 0.01 | <.001 | 0.32 to 0.36 | | 0.34 | 0.01 | <.001 | 0.32 to 0.36 | |
| Finland (ref.) |  |  |  |  |  |  |  |  |  |  |
| France | 1.68 | 0.13 | <.001 | 1.43 to 1.93 | | 1.68 | 0.13 | <.001 | 1.43 to 1.93 | |
| Germany | 0.53 | 0.13 | <.001 | 0.27 to 0.78 | | 0.53 | 0.13 | <.001 | 0.27 to 0.78 | |
| Ireland | 0.68 | 0.14 | <.001 | 0.40 to 0.96 | | 0.68 | 0.14 | <.001 | 0.40 to 0.96 | |
| Italy | 1.41 | 0.13 | <.001 | 1.15 to 1.66 | | 1.41 | 0.13 | <.001 | 1.15 to 1.66 | |
| Poland | 0.51 | 0.13 | <.001 | 0.25 to 0.76 | | 0.51 | 0.13 | <.001 | 0.25 to 0.76 | |
| Finland * w_wtech (ref.) |  |  |  |  |  |  |  |  |  |  |
| France * w_wtech |  |  |  |  |  | -0.04 | 0.06 | 0.461 | -0.16 to 0.07 | |
| Germany * w_wtech |  |  |  |  |  | -0.02 | 0.06 | 0.732 | -0.14 to 0.10 | |
| Ireland * w_wtech |  |  |  |  |  | -0.02 | 0.07 | 0.819 | -0.15 to 0.12 | |
| Italy * w_wtech |  |  |  |  |  | 0.03 | 0.05 | 0.603 | -0.08 to 0.13 | |
| Poland * w_wtech |  |  |  |  |  | 0.01 | 0.05 | 0.884 | -0.10 to 0.11 | |
| Competence frustration | M1 | | | | | M2 | | | | |
| variables | B | SE | *P* | 95% CI | | B | SE | *P* | 95% CI | |
| w_wtech | 0.07 | 0.02 | <.001 | 0.04 to 0.11 | | 0.06 | 0.04 | 0.173 | -0.03 to 0.14 | |
| b_wtech | 0.16 | 0.01 | <.001 | 0.13 to 0.18 | | 0.16 | 0.01 | <.001 | 0.13 to 0.18 | |
| Finland (ref.) |  |  |  |  |  |  |  |  |  |  |
| France | 1.82 | 0.14 | <.001 | 1.55 to 2.09 | | 1.82 | 0.14 | <.001 | 1.55 to 2.09 | |
| Germany | 0.37 | 0.14 | 0.009 | 0.09 to 0.64 | | 0.37 | 0.14 | 0.009 | 0.09 to 0.64 | |
| Ireland | 0.20 | 0.16 | 0.191 | -0.10 to 0.51 | | 0.20 | 0.16 | 0.191 | -0.10 to 0.51 | |
| Italy | 1.58 | 0.14 | <.001 | 1.31 to 1.86 | | 1.58 | 0.14 | <.001 | 1.31 to 1.86 | |
| Poland | 1.04 | 0.14 | <.001 | 0.77 to 1.32 | | 1.04 | 0.14 | <.001 | 0.77 to 1.32 | |
| Finland * w_wtech (ref.) |  |  |  |  |  |  |  |  |  |  |
| France * w_wtech |  |  |  |  |  | -0.02 | 0.06 | 0.745 | -0.14 to 0.10 | |
| Germany * w_wtech |  |  |  |  |  | 0.03 | 0.07 | 0.648 | -0.10 to 0.16 | |
| Ireland * w_wtech |  |  |  |  |  | -0.05 | 0.07 | 0.490 | -0.19 to 0.09 | |
| Italy * w_wtech |  |  |  |  |  | 0.08 | 0.06 | 0.135 | -0.03 to 0.19 | |
| Poland * w_wtech |  |  |  |  |  | -0.02 | 0.06 | 0.755 | -0.13 to 0.09 | |
| Relatedness satisfaction | M1 | | | | | M2 | | | | |
| Variables | B | SE | *P* | 95% CI | | B | SE | *P* | 95% CI | |
| w_wtech | 0.24 | 0.02 | <.001 | 0.21 to 0.27 | | 0.21 | 0.04 | <.001 | 0.12 to 0.29 | |
| b_wtech | 0.56 | 0.01 | <.001 | 0.53 to 0.58 | | 0.56 | 0.01 | <.001 | 0.53 to 0.58 | |
| Finland (ref.) |  |  |  |  |  |  |  |  |  |  |
| France | 1.20 | 0.14 | <.001 | 0.94 to 1.47 | | 1.20 | 0.14 | <.001 | 0.94 to 1.47 | |
| Germany | 0.14 | 0.14 | 0.326 | -0.14 to 0.41 | | 0.14 | 0.14 | 0.326 | -0.14 to 0.41 | |
| Ireland | 0.41 | 0.15 | 0.007 | 0.11 to 0.71 | | 0.41 | 0.15 | 0.007 | 0.11 to 0.71 | |
| Italy | 2.68 | 0.14 | <.001 | 2.41 to 2.94 | | 2.68 | 0.14 | <.001 | 2.41 to 2.94 | |
| Poland | 0.45 | 0.14 | 0.001 | 0.18 to 0.72 | | 0.45 | 0.14 | 0.001 | 0.18 to 0.72 | |
| Finland * w_wtech (ref.) |  |  |  |  |  |  |  |  |  |  |
| France * w_wtech |  |  |  |  |  | 0.02 | 0.06 | 0.733 | -0.10 to 0.14 | |
| Germany * w_wtech |  |  |  |  |  | -0.02 | 0.07 | 0.820 | -0.14 to 0.11 | |
| Ireland * w_wtech |  |  |  |  |  | 0.01 | 0.07 | 0.883 | -0.13 to 0.15 | |
| Italy * w_wtech |  |  |  |  |  | 0.06 | 0.06 | 0.246 | -0.04 to 0.17 | |
| Poland * w_wtech |  |  |  |  |  | 0.06 | 0.06 | 0.317 | -0.05 to 0.16 | |
