# Supplementary material for: Use of Health and Well-Being Technology, Basic Psychological Needs, and the Mediating Role of Technological Identity in 6 European Countries: Prospective Longitudinal Survey Study
Source: J Med Internet Res. 2026 May 19;28:e83054. doi: 10.2196/83054 (PMC13231114; doi:10.2196/83054)
Supplement: Multimedia Appendix 6 [file jmir_v28i1e83054_app6.docx]

|  | Model 3 | | | | | Model 4 | | | | | Model 5 | | | | |
| --- | --- | --- | --- | --- | --- | --- | --- | --- | --- | --- | --- | --- | --- | --- | --- |
| Autonomy frustration | β | SE | *P* | 95% CI | | β | SE | *P* | 95% CI | | β | SE | *P* | 95% CI | |
| Autonomy lag | 0.06 | 0.03 | 0.022 | 0.01 to 0.11 | | 0.06 | 0.02 | 0.010 | 0.02 to 0.11 | | 0.06 | 0.02 | 0.010 | 0.02 to 0.11 | |
| Wtech | 0.06 | 0.02 | 0.011 | 0.01 to 0.10 | | 0.06 | 0.02 | 0.006 | 0.02 to 0.10 | | 0.05 | 0.02 | 0.017 | 0.01 to 0.10 | |
| Wtech lag | -0.01 | 0.02 | 0.567 | -0.05 to 0.03 | |  |  |  |  |  | -0.01 | 0.02 | 0.589 | -0.05 to 0.03 | |
| Finland (ref.) |  |  |  |  |  |  |  |  |  |  |  |  |  |  |  |
| France | 0.12 | 0.01 | <.001 | 0.09 to 0.14 | | 0.12 | 0.01 | <.001 | 0.10 to 0.15 | | 0.12 | 0.01 | <.001 | 0.09 to 0.15 | |
| Germany | -0.01 | 0.01 | 0.375 | -0.04 to 0.01 | | -0.01 | 0.01 | 0.683 | -0.03 to 0.02 | | -0.01 | 0.01 | 0.665 | -0.03 to 0.02 | |
| Ireland | 0.04 | 0.01 | 0.004 | 0.01 to 0.06 | | 0.03 | 0.01 | 0.019 | 0.01 to 0.06 | | 0.03 | 0.01 | 0.020 | 0.01 to 0.06 | |
| Italy | 0.12 | 0.01 | <.001 | 0.10 to 0.15 | | 0.11 | 0.01 | <.001 | 0.09 to 0.14 | | 0.11 | 0.01 | <.001 | 0.09 to 0.14 | |
| Poland | 0.04 | 0.01 | 0.002 | 0.02 to 0.07 | | 0.03 | 0.01 | 0.029 | 0.00 to 0.05 | | 0.03 | 0.01 | 0.029 | 0.00 to 0.06 | |
| Self-esteem |  |  |  |  |  | 0.00 | 0.02 | 0.905 | -0.04 to 0.04 | | 0.00 | 0.02 | 0.896 | -0.04 to 0.04 | |
| SCO |  |  |  |  |  | 0.05 | 0.02 | 0.003 | 0.02 to 0.09 | | 0.05 | 0.02 | 0.003 | 0.02 to 0.09 | |
| Self-rated health |  |  |  |  |  | -0.01 | 0.02 | 0.760 | -0.05 to 0.03 | | -0.01 | 0.02 | 0.764 | -0.05 to 0.03 | |
| Happiness |  |  |  |  |  | 0.01 | 0.02 | 0.732 | -0.03 to 0.04 | | 0.01 | 0.02 | 0.735 | -0.03 to 0.04 | |
| Male |  |  |  |  |  | 0.09 | 0.01 | <.001 | 0.07 to 0.11 | | 0.09 | 0.01 | <.001 | 0.07 to 0.11 | |
| Age |  |  |  |  |  | -0.15 | 0.01 | <.001 | -0.18 to -0.13 | | -0.16 | 0.02 | <.001 | -0.19 to -0.13 | |
| Education |  |  |  |  |  | 0.03 | 0.01 | 0.001 | 0.01 to 0.05 | | 0.04 | 0.01 | 0.001 | 0.01 to 0.06 | |
| Competence frustration | β | SE | *P* | 95% CI | | β | SE | *P* | 95% CI | | β | SE | *P* | 95% CI | |
| Competence lag | 0.09 | 0.03 | 0.004 | 0.03 to 0.15 | | 0.09 | 0.03 | 0.003 | 0.03 to 0.14 | | 0.08 | 0.03 | 0.004 | 0.03 to 0.14 | |
| Wtech | 0.06 | 0.02 | 0.008 | 0.02 to 0.11 | | 0.06 | 0.02 | 0.011 | 0.01 to 0.10 | | 0.06 | 0.02 | 0.010 | 0.01 to 0.11 | |
| Wtech lag | 0.01 | 0.02 | 0.604 | -0.03 to 0.06 | |  |  |  |  |  | 0.01 | 0.02 | 0.600 | -0.03 to 0.06 | |
| Finland (ref.) |  |  |  |  |  |  |  |  |  |  |  |  |  |  |  |
| France | 0.14 | 0.01 | <.001 | 0.11 to 0.16 | | 0.14 | 0.01 | <.001 | 0.11 to 0.16 | | 0.14 | 0.02 | <.001 | 0.11 to 0.17 | |
| Germany | 0.01 | 0.01 | 0.343 | -0.01 to 0.04 | | 0.02 | 0.01 | 0.251 | -0.01 to 0.04 | | 0.02 | 0.01 | 0.220 | -0.01 to 0.04 | |
| Ireland | 0.01 | 0.01 | 0.541 | -0.02 to 0.03 | | 0.01 | 0.01 | 0.596 | -0.02 to 0.03 | | 0.01 | 0.01 | 0.624 | -0.02 to 0.03 | |
| Italy | 0.13 | 0.01 | <.001 | 0.10 to 0.15 | | 0.12 | 0.01 | <.001 | 0.09 to 0.15 | | 0.12 | 0.01 | <.001 | 0.09 to 0.15 | |
| Poland | 0.09 | 0.01 | <.001 | 0.06 to 0.11 | | 0.08 | 0.01 | <.001 | 0.05 to 0.11 | | 0.08 | 0.01 | <.001 | 0.05 to 0.11 | |
| Self-esteem |  |  |  |  |  | -0.01 | 0.02 | 0.504 | -0.05 to 0.03 | | -0.01 | 0.02 | 0.504 | -0.05 to 0.03 | |
| SCO |  |  |  |  |  | 0.04 | 0.02 | 0.040 | 0.00 to 0.08 | | 0.04 | 0.02 | 0.040 | 0.00 to 0.08 | |
| Self-rated health |  |  |  |  |  | -0.02 | 0.02 | 0.417 | -0.06 to 0.02 | | -0.02 | 0.02 | 0.413 | -0.06 to 0.02 | |
| Happiness |  |  |  |  |  | -0.01 | 0.02 | 0.610 | -0.04 to 0.03 | | -0.01 | 0.02 | 0.611 | -0.04 to 0.03 | |
| Male |  |  |  |  |  | 0.02 | 0.01 | 0.081 | 0.00 to 0.04 | | 0.02 | 0.01 | 0.096 | -0.00 to 0.04 | |
| Age |  |  |  |  |  | -0.09 | 0.01 | <.001 | -0.12 to -0.06 | | -0.09 | 0.02 | <.001 | -0.12 to -0.06 | |
| Education |  |  |  |  |  | 0.00 | 0.01 | 0.871 | -0.02 to 0.02 | | 0.00 | 0.01 | 0.993 | -0.02 to 0.02 | |
| Relatedness satisfaction | β | SE | *P* | CI | | β | SE | *P* | 95% CI | | β | SE | *P* | 95% CI | |
| Relatedness lag | 0.05 | 0.03 | 0.070 | 0.00 to 0.10 | | 0.04 | 0.02 | 0.088 | -0.01 to 0.09 | | 0.05 | 0.03 | 0.069 | -0.00 to 0.09 | |
| Wtech | 0.14 | 0.02 | <.001 | 0.10 to 0.18 | | 0.14 | 0.02 | <.001 | 0.10 to 0.18 | | 0.14 | 0.02 | <.001 | 0.10 to 0.18 | |
| Wtech lag | -0.02 | 0.02 | 0.410 | -0.06 to 0.02 | |  |  |  |  |  | -0.02 | 0.020 | 0.432 | -0.05 to 0.02 | |
| Finland (ref.) |  |  |  |  |  |  |  |  |  |  |  |  |  |  |  |
| France | 0.09 | 0.01 | <.001 | 0.06 to 0.11 | | 0.09 | 0.01 | <.001 | 0.07 to 0.12 | | 0.09 | 0.01 | <.001 | 0.07 to 0.12 | |
| Germany | -0.01 | 0.01 | 0.597 | -0.03 to 0.02 | | 0.00 | 0.012 | 0.941 | -0.03 to 0.02 | | 0.00 | 0.01 | 0.893 | -0.03 to 0.02 | |
| Ireland | 0.04 | 0.01 | 0.004 | 0.01 to 0.06 | | 0.03 | 0.01 | 0.021 | 0.00 to 0.05 | | 0.03 | 0.01 | 0.020 | 0.01 to 0.05 | |
| Italy | 0.23 | 0.01 | <.001 | 0.20 to 0.26 | | 0.22 | 0.01 | <.001 | 0.19 to 0.24 | | 0.22 | 0.01 | <.001 | 0.19 to 0.24 | |
| Poland | 0.06 | 0.01 | <.001 | 0.04 to 0.09 | | 0.05 | 0.01 | <.001 | 0.03 to 0.08 | | 0.05 | 0.01 | <.001 | 0.03 to 0.08 | |
| Self-esteem |  |  |  |  |  | 0.02 | 0.02 | 0.263 | -0.02 to 0.06 | | 0.02 | 0.02 | 0.266 | -0.02 to 0.06 | |
| SCO |  |  |  |  |  | 0.07 | 0.02 | <.001 | 0.04 to 0.10 | | 0.07 | 0.02 | <.001 | 0.04 to 0.10 | |
| Self-rated health |  |  |  |  |  | 0.00 | 0.02 | 0.901 | -0.04 to 0.03 | | 0.00 | 0.02 | 0.911 | -0.04 to 0.03 | |
| Happiness |  |  |  |  |  | -0.01 | 0.02 | 0.587 | -0.04 to 0.02 | | -0.01 | 0.02 | 0.581 | -0.04 to 0.02 | |
| Male |  |  |  |  |  | 0.06 | 0.01 | <.001 | 0.05 to 0.08 | | 0.06 | 0.01 | <.001 | 0.05 to 0.08 | |
| Age |  |  |  |  |  | -0.09 | 0.01 | <.001 | -0.11 to -0.06 | | -0.09 | 0.01 | <.001 | -0.12 to -0.06 | |
| Education |  |  |  |  |  | 0.03 | 0.01 | 0.004 | 0.01 to 0.05 | | 0.03 | 0.01 | 0.003 | 0.01 to 0.05 | |
